# Tetra-μ-acetato-κ^8^ *O*:*O*′-bis­[(3-cyano­pyridine-κ*N* ^1^)ruthenium(II,III)](*Ru*—*Ru*) hexa­fluoridophosphate 1,2-dichloro­ethane monosolvate

**DOI:** 10.1107/S1600536811041997

**Published:** 2011-10-22

**Authors:** Samuel A. Minaker, Ruiyao Wang, Manuel A. S. Aquino

**Affiliations:** a Department of Chemistry, St Francis Xavier University, PO Box 5000, Antigonish, Nova Scotia, Canada B2G 2W5; b Department of Chemistry, Queen’s University, Kingston, Ontario, Canada K7L 3N6

## Abstract

The title compound, [Ru_2_(CH_3_CO_2_)_4_(C_6_H_4_N_2_)_2_]PF_6_·C_2_H_4_Cl_2_, was obtained *via* a rapid substitution reaction in 2-propanol whereby 3-cyano­pyridine replaces the axial water mol­ecules in the diaquatetra-μ-acetato-diruthenium(II,III) hexa­fluorido­phosphate starting material. The product rapidly precipated and crystals were grown from 1,2-dichloro­ethane. The 1,2-dichloro­ethane mol­ecule of solvation exhibits disorder with two different orientations [occupancy ratio 0.51 (6):0.49 (6)]. All three parts, the cation, the anion and the disordered solvent mol­ecule lie on crystallographic inversion centers. The Ru—Ru bond length of 2.2702 (6) Å fits nicely into the range seen for similar complexes and correlates well with the reduction potential of the complex and donor strength of the axial ligand, 3-cyano­pyridine, as postulated in a previous study [Vamvounis *et al.* (2000 ▶). *Inorg. Chim. Acta*, **305**, 87–98]. The 3-cyano­pyridine ligands orient themselves in an *anti* configuration with respect to each other and the Ru—Ru—N angle [174.27 (7)°] is close to being linear.

## Related literature

For related structures and physical measurements, see: Vamvounis *et al.* (2000 ▶).
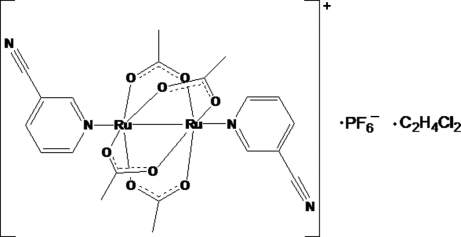


## Experimental

### 

#### Crystal data

[Ru_2_(C_2_H_3_O_2_)_4_(C_6_H_4_N_2_)_2_]PF_6_·C_2_H_4_Cl_2_
*M* *_r_* = 890.46
Triclinic, 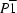

*a* = 8.1743 (6) Å
*b* = 10.3955 (10) Å
*c* = 11.397 (1) Å
α = 105.860 (6)°
β = 108.929 (5)°
γ = 104.099 (5)°
*V* = 820.38 (14) Å^3^
*Z* = 1
Mo *K*α radiation
μ = 1.21 mm^−1^
*T* = 180 K
0.20 × 0.20 × 0.15 mm

#### Data collection

Bruker APEXII CCD diffractometer
Absorption correction: multi-scan (*SADABS*; Bruker, 2010) ▶ *T*_min_ = 0.793, *T*_max_ = 0.839
6133 measured reflections
3175 independent reflections
2784 reflections with *I* > 2σ(*I*)
*R*_int_ = 0.027

#### Refinement

*R*[*F* ^2^ > 2σ(*F* ^2^)] = 0.028
*wR*(*F* ^2^) = 0.078
*S* = 1.09
3175 reflections
226 parameters
H-atom parameters constrained
Δρ_max_ = 0.85 e Å^−3^
Δρ_min_ = −0.76 e Å^−3^

### 

Data collection: *APEX2* (Bruker, 2010 ▶); cell refinement: *SAINT* (Bruker, 2010 ▶); data reduction: *SAINT*; program(s) used to solve structure: *SHELXTL* (Sheldrick, 2008 ▶); program(s) used to refine structure: *SHELXTL*; molecular graphics: *SHELXTL*; software used to prepare material for publication: *SHELXTL*.

## Supplementary Material

Crystal structure: contains datablock(s) I, global. DOI: 10.1107/S1600536811041997/nk2113sup1.cif

Structure factors: contains datablock(s) I. DOI: 10.1107/S1600536811041997/nk2113Isup2.hkl

Additional supplementary materials:  crystallographic information; 3D view; checkCIF report

## Figures and Tables

**Fig. 1.**
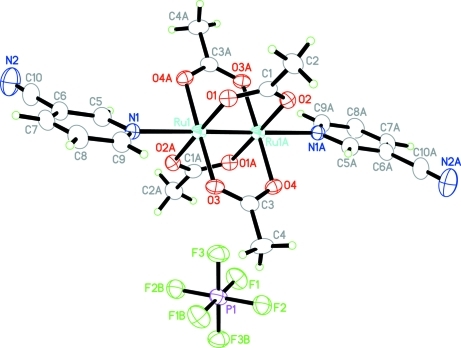
The molecular structure of (I), with atom labels and 50% probability ellipsoids for the non-H atoms. Solvent molecule omitted for clarity. Symmetry operator A = -x + 1, -y + 1, -z + 1; B = -x, -y + 1, -z.

## supplementary crystallographic information

### Comment

A number of years ago our lab synthesized and structurally characterized a series of diruthenium(II,III) tetraacetate complexes with different axial donor ligands of varying donor strengths (Vamvounis *et al.*, 2000). One reason to study these was to synthesize dimers with asymmetric bidendate axial donors that could act as bridges for mixed-metal metallopolymers and extended arrays (*i.e.* in the case of cyanopyridine adducts the pyridine end could be coordinated to a harder metal than the cyano end). The other reason was to correlate axial donor strength (as well as redox potential) with the Ru—Ru bond length. These ligands ranged from weak donors such as water and methanol to relatively strong donors such as dimethylformamide, dimethylsulfoxide and various pyridine derivatives. Unfortunately while we were able to structurally characterize the 4-cyanopyridine adduct in the earlier paper we were unable to obtain the 3-cyanopyridine adduct. This structure is now finally reported here.

The title compound (I) (Fig. 1) can be compared to the 4-cyanopyridine adduct reported previously (Vamvounis *et al.*, 2000). The Ru—Ru bond lengths are 2.2702 (6) Å and 2.2741 (7) Å respectively which fits well into correlation of Ru—Ru bond length with axial ligand donor strength as outlined in the earlier paper. (*i.e.* the 3-cyanopyridine being the slightly weaker donor as measured electrochemically manifests a shorter Ru—Ru bond length structurally in the complex because less electron density is being donated into the metal-metal antibonding HOMO). The 3-cyanopyridine ligands are situated *anti* with respect to each other and the pyridine planes essentially bifurcate the planes formed by the perpendicular carboxylate groups (O—C—O), *e.g*. the O1—Ru1—N1—C5 torsion angle is -48.5 °.

### Experimental

The method of preparation of the title compound (I) was similar to the method used by (Vamvounis *et al.*, 2000) in preparing the earlier pyridine adducts of diruthenium(II,III) tetraacetate except that a 2.1:1 ligand to metal ratio was used instead of a 4:1 ratio. For example, [Ru_2_(µ-O_2_CCH_3_)_4_(H_2_O)_2_](PF_6_) (0.100 g, 0.161 mmol) was dissolved in 10 ml of 2-propanol. A 2.1-fold access of 3-cyanopyridine (0.037 g, 0.338 mmol) was added with stirring and a green precipitate formed immediately. The solution was stirred for another 5 minutes and the olive-green product collected *via* suction filtration, washed with 50 ml of 2-propanol and dried *in vacuo*. (Yield = 0.101 g, 79%). Crystals were grown by slow evaporation from 1,2-dichloroethane.

### Refinement

The structure was solved by direct methods. All non-hydrogen atoms were refined anisotropically. All H atoms were placed in geometrically calculated positions, with C—H = 0.95 (aromatic), 0.99(CH_2_) and 0.98 (methyl) Å, and refined as riding atoms, with *U*_iso_(H) = 1.5 *U*_eq_(C) (methyl), and 1.2 *U*_eq_(other C). In addition, the methyl groups were refined with AFIX 137, which allowed the rotation of the methyl groups whilst keeping the C—H distances and *X*—C—H angles fixed. The solvent molecule C_2_H_4_Cl_2_ in the structure is disordered. It was split and refined into two parts with different orientations and with nearly equal occupancies.

### Crystal data

**Table:**

[Ru_2_(C_2_H_3_O_2_)_4_(C_6_H_4_N_2_)_2_]PF_6_·C_2_H_4_Cl_2_	*Z* = 1
*M**_r_* = 890.46	*F*(000) = 439
Triclinic, *P*1	*D*_x_ = 1.802 Mg m^−^^3^
Hall symbol: -P 1	Mo *K*α radiation, λ = 0.71073 Å
*a* = 8.1743 (6) Å	Cell parameters from 4068 reflections
*b* = 10.3955 (10) Å	θ = 2.4–27.1°
*c* = 11.397 (1) Å	µ = 1.21 mm^−^^1^
α = 105.860 (6)°	*T* = 180 K
β = 108.929 (5)°	Block, brown
γ = 104.099 (5)°	0.20 × 0.20 × 0.15 mm
*V* = 820.38 (14) Å^3^	

### Data collection

**Table:**

Bruker APEXII CCD diffractometer	3175 independent reflections
Radiation source: fine-focus sealed tube	2784 reflections with *I* > 2σ(*I*)
graphite	*R*_int_ = 0.027
φ and ω scans	θ_max_ = 26.0°, θ_min_ = 2.1°
Absorption correction: multi-scan (*SADABS*; Bruker, 2010)	*h* = −10→10
*T*_min_ = 0.793, *T*_max_ = 0.839	*k* = −12→12
6133 measured reflections	*l* = −14→12

### Refinement

**Table:**

Refinement on *F*^2^	Primary atom site location: structure-invariant direct methods
Least-squares matrix: full	Secondary atom site location: difference Fourier map
*R*[*F*^2^ > 2σ(*F*^2^)] = 0.028	Hydrogen site location: inferred from neighbouring sites
*wR*(*F*^2^) = 0.078	H-atom parameters constrained
*S* = 1.09	*w* = 1/[σ^2^(*F*_o_^2^) + (0.0226*P*)^2^ + 1.188*P*] where *P* = (*F*_o_^2^ + 2*F*_c_^2^)/3
3175 reflections	(Δ/σ)_max_ < 0.001
226 parameters	Δρ_max_ = 0.85 e Å^−^^3^
0 restraints	Δρ_min_ = −0.76 e Å^−^^3^

### Special details

**Table:**

Geometry. All e.s.d.'s (except the e.s.d. in the dihedral angle between two l.s. planes) are estimated using the full covariance matrix. The cell e.s.d.'s are taken into account individually in the estimation of e.s.d.'s in distances, angles and torsion angles; correlations between e.s.d.'s in cell parameters are only used when they are defined by crystal symmetry. An approximate (isotropic) treatment of cell e.s.d.'s is used for estimating e.s.d.'s involving l.s. planes.
Refinement. Refinement of *F*^2^ against ALL reflections. The weighted *R*-factor *wR* and goodness of fit *S* are based on *F*^2^, conventional *R*-factors *R* are based on *F*, with *F* set to zero for negative *F*^2^. The threshold expression of *F*^2^ > σ(*F*^2^) is used only for calculating *R*-factors(gt) *etc*. and is not relevant to the choice of reflections for refinement. *R*-factors based on *F*^2^ are statistically about twice as large as those based on *F*, and *R*- factors based on ALL data will be even larger.

### Fractional atomic coordinates and isotropic or equivalent isotropic displacement parameters (Å^2^)

**Table:**

	*x*	*y*	*z*	*U*_iso_*/*U*_eq_	Occ. (<1)
P1	0.0000	0.5000	0.0000	0.0346 (3)	
Ru1	0.47865 (3)	0.38173 (3)	0.45928 (2)	0.02328 (10)	
O1	0.7543 (3)	0.4309 (2)	0.5531 (2)	0.0269 (5)	
O2	0.7964 (3)	0.6638 (2)	0.6343 (2)	0.0270 (5)	
O3	0.5095 (3)	0.3989 (2)	0.2954 (2)	0.0271 (5)	
O4	0.5481 (3)	0.6314 (2)	0.3736 (2)	0.0270 (5)	
N1	0.4058 (4)	0.1379 (3)	0.3754 (3)	0.0256 (6)	
N2	−0.0128 (5)	−0.2151 (4)	0.4373 (4)	0.0600 (10)	
C1	0.8583 (4)	0.5624 (4)	0.6211 (3)	0.0274 (7)	
C2	1.0631 (4)	0.6004 (4)	0.6875 (4)	0.0359 (8)	
H2A	1.1126	0.6717	0.7792	0.054*	
H2B	1.1218	0.6405	0.6364	0.054*	
H2C	1.0893	0.5140	0.6906	0.054*	
C3	0.5368 (4)	0.5203 (4)	0.2848 (3)	0.0272 (7)	
C4	0.5549 (5)	0.5324 (4)	0.1623 (4)	0.0373 (8)	
H4A	0.6669	0.6146	0.1884	0.056*	
H4B	0.4457	0.5463	0.1073	0.056*	
H4C	0.5640	0.4443	0.1104	0.056*	
C5	0.2756 (5)	0.0610 (4)	0.4029 (4)	0.0345 (8)	
H5A	0.2130	0.1083	0.4463	0.041*	
C6	0.2307 (4)	−0.0857 (4)	0.3693 (4)	0.0324 (7)	
C7	0.3223 (5)	−0.1549 (4)	0.3063 (4)	0.0347 (8)	
H7A	0.2967	−0.2547	0.2849	0.042*	
C8	0.4511 (5)	−0.0752 (4)	0.2759 (4)	0.0391 (8)	
H8A	0.5140	−0.1197	0.2307	0.047*	
C9	0.4882 (5)	0.0701 (4)	0.3115 (4)	0.0341 (8)	
H9A	0.5768	0.1239	0.2891	0.041*	
C10	0.0933 (5)	−0.1605 (4)	0.4058 (4)	0.0411 (9)	
F1	−0.0541 (3)	0.6132 (3)	0.0917 (2)	0.0518 (6)	
F2	0.1696 (3)	0.6283 (3)	0.0146 (2)	0.0474 (6)	
F3	0.1334 (3)	0.4879 (3)	0.1314 (2)	0.0485 (6)	
Cl1A	0.242 (4)	0.032 (3)	0.9565 (15)	0.163 (4)	0.51 (6)
C11A	0.056 (5)	0.077 (4)	1.010 (4)	0.108 (10)	0.51 (6)
H11A	0.1094	0.1457	1.1040	0.129*	0.51 (6)
H11B	−0.0193	0.1142	0.9496	0.129*	0.51 (6)
Cl1B	0.191 (4)	0.0453 (18)	0.914 (4)	0.152 (7)	0.49 (6)
C11B	0.013 (10)	−0.052 (7)	0.969 (5)	0.21 (3)	0.49 (6)
H11C	−0.1020	−0.1225	0.8912	0.246*	0.49 (6)
H11D	0.0663	−0.0995	1.0277	0.246*	0.49 (6)

### Atomic displacement parameters (Å^2^)

**Table:**

	*U*^11^	*U*^22^	*U*^33^	*U*^12^	*U*^13^	*U*^23^
P1	0.0257 (6)	0.0448 (8)	0.0286 (7)	0.0091 (6)	0.0114 (5)	0.0107 (6)
Ru1	0.02188 (14)	0.02672 (16)	0.02337 (15)	0.00927 (11)	0.01160 (11)	0.00975 (11)
O1	0.0250 (11)	0.0321 (13)	0.0288 (12)	0.0144 (10)	0.0142 (10)	0.0121 (10)
O2	0.0245 (11)	0.0280 (12)	0.0279 (12)	0.0087 (9)	0.0122 (9)	0.0093 (10)
O3	0.0275 (11)	0.0353 (13)	0.0221 (11)	0.0143 (10)	0.0128 (9)	0.0108 (10)
O4	0.0256 (11)	0.0331 (13)	0.0282 (12)	0.0126 (10)	0.0137 (10)	0.0157 (10)
N1	0.0284 (14)	0.0222 (13)	0.0245 (14)	0.0091 (11)	0.0094 (11)	0.0086 (11)
N2	0.056 (2)	0.047 (2)	0.086 (3)	0.0132 (18)	0.044 (2)	0.027 (2)
C1	0.0251 (16)	0.0387 (19)	0.0245 (16)	0.0123 (15)	0.0148 (13)	0.0150 (15)
C2	0.0217 (16)	0.044 (2)	0.039 (2)	0.0108 (15)	0.0114 (15)	0.0139 (17)
C3	0.0192 (15)	0.0380 (19)	0.0258 (16)	0.0125 (14)	0.0101 (13)	0.0117 (15)
C4	0.0389 (19)	0.055 (2)	0.0297 (19)	0.0225 (18)	0.0203 (16)	0.0222 (18)
C5	0.0313 (18)	0.0354 (19)	0.037 (2)	0.0124 (15)	0.0161 (16)	0.0123 (16)
C6	0.0256 (16)	0.0301 (18)	0.0351 (19)	0.0069 (14)	0.0086 (15)	0.0111 (15)
C7	0.0350 (19)	0.0246 (17)	0.0357 (19)	0.0116 (15)	0.0093 (15)	0.0049 (15)
C8	0.041 (2)	0.036 (2)	0.042 (2)	0.0174 (17)	0.0242 (18)	0.0055 (17)
C9	0.0359 (19)	0.0325 (19)	0.0364 (19)	0.0144 (15)	0.0200 (16)	0.0094 (16)
C10	0.035 (2)	0.033 (2)	0.052 (2)	0.0089 (16)	0.0180 (18)	0.0144 (18)
F1	0.0460 (13)	0.0548 (15)	0.0486 (14)	0.0190 (11)	0.0235 (11)	0.0064 (12)
F2	0.0361 (12)	0.0529 (14)	0.0419 (13)	0.0030 (10)	0.0152 (10)	0.0151 (11)
F3	0.0350 (12)	0.0666 (16)	0.0383 (13)	0.0138 (11)	0.0092 (10)	0.0239 (12)
Cl1A	0.185 (10)	0.204 (9)	0.091 (6)	0.089 (8)	0.039 (6)	0.050 (5)
C11A	0.15 (2)	0.068 (16)	0.075 (12)	0.049 (13)	0.014 (13)	0.018 (11)
Cl1B	0.132 (9)	0.179 (7)	0.158 (15)	0.066 (6)	0.068 (9)	0.067 (8)
C11B	0.20 (5)	0.18 (5)	0.11 (3)	0.06 (4)	0.00 (3)	−0.03 (2)

### Geometric parameters (Å, °)

**Table:**

P1—F1^i^	1.598 (2)	C2—H2B	0.9800
P1—F1	1.598 (2)	C2—H2C	0.9800
P1—F2	1.599 (2)	C3—C4	1.485 (4)
P1—F2^i^	1.599 (2)	C4—H4A	0.9800
P1—F3	1.600 (2)	C4—H4B	0.9800
P1—F3^i^	1.600 (2)	C4—H4C	0.9800
Ru1—O3	2.012 (2)	C5—C6	1.387 (5)
Ru1—O1	2.015 (2)	C5—H5A	0.9500
Ru1—O2^ii^	2.016 (2)	C6—C7	1.387 (5)
Ru1—O4^ii^	2.023 (2)	C6—C10	1.447 (5)
Ru1—Ru1^ii^	2.2702 (6)	C7—C8	1.373 (5)
Ru1—N1	2.295 (3)	C7—H7A	0.9500
O1—C1	1.273 (4)	C8—C9	1.378 (5)
O2—C1	1.270 (4)	C8—H8A	0.9500
O2—Ru1^ii^	2.016 (2)	C9—H9A	0.9500
O3—C3	1.273 (4)	Cl1A—C11A	1.93 (4)
O4—C3	1.272 (4)	C11A—C11A^iii^	1.56 (7)
O4—Ru1^ii^	2.023 (2)	C11A—H11A	0.9900
N1—C9	1.323 (4)	C11A—H11B	0.9900
N1—C5	1.345 (4)	Cl1B—C11B	1.93 (8)
N2—C10	1.130 (5)	C11B—C11B^iii^	1.22 (11)
C1—C2	1.491 (4)	C11B—H11C	0.9900
C2—H2A	0.9800	C11B—H11D	0.9900
			
F1^i^—P1—F1	180.00 (16)	H2A—C2—H2B	109.5
F1^i^—P1—F2	89.90 (12)	C1—C2—H2C	109.5
F1—P1—F2	90.10 (12)	H2A—C2—H2C	109.5
F1^i^—P1—F2^i^	90.11 (12)	H2B—C2—H2C	109.5
F1—P1—F2^i^	89.89 (12)	O4—C3—O3	123.1 (3)
F2—P1—F2^i^	180.0	O4—C3—C4	118.5 (3)
F1^i^—P1—F3	90.18 (13)	O3—C3—C4	118.5 (3)
F1—P1—F3	89.82 (13)	C3—C4—H4A	109.5
F2—P1—F3	89.81 (12)	C3—C4—H4B	109.5
F2^i^—P1—F3	90.19 (12)	H4A—C4—H4B	109.5
F1^i^—P1—F3^i^	89.82 (13)	C3—C4—H4C	109.5
F1—P1—F3^i^	90.18 (13)	H4A—C4—H4C	109.5
F2—P1—F3^i^	90.19 (12)	H4B—C4—H4C	109.5
F2^i^—P1—F3^i^	89.81 (12)	N1—C5—C6	121.5 (3)
F3—P1—F3^i^	180.0	N1—C5—H5A	119.3
O3—Ru1—O1	89.72 (9)	C6—C5—H5A	119.3
O3—Ru1—O2^ii^	89.97 (9)	C7—C6—C5	119.4 (3)
O1—Ru1—O2^ii^	178.99 (9)	C7—C6—C10	122.2 (3)
O3—Ru1—O4^ii^	178.79 (9)	C5—C6—C10	118.4 (3)
O1—Ru1—O4^ii^	89.53 (9)	C8—C7—C6	118.1 (3)
O2^ii^—Ru1—O4^ii^	90.77 (8)	C8—C7—H7A	120.9
O3—Ru1—Ru1^ii^	90.35 (7)	C6—C7—H7A	120.9
O1—Ru1—Ru1^ii^	90.07 (7)	C7—C8—C9	119.4 (3)
O2^ii^—Ru1—Ru1^ii^	88.97 (6)	C7—C8—H8A	120.3
O4^ii^—Ru1—Ru1^ii^	88.71 (7)	C9—C8—H8A	120.3
O3—Ru1—N1	92.05 (9)	N1—C9—C8	122.8 (3)
O1—Ru1—N1	95.14 (9)	N1—C9—H9A	118.6
O2^ii^—Ru1—N1	85.83 (9)	C8—C9—H9A	118.6
O4^ii^—Ru1—N1	88.95 (9)	N2—C10—C6	177.9 (4)
Ru1^ii^—Ru1—N1	174.27 (7)	C11A^iii^—C11A—Cl1A	97 (3)
C1—O1—Ru1	118.3 (2)	C11A^iii^—C11A—H11A	112.3
C1—O2—Ru1^ii^	119.4 (2)	Cl1A—C11A—H11A	112.3
C3—O3—Ru1	118.4 (2)	C11A^iii^—C11A—H11B	112.3
C3—O4—Ru1^ii^	119.5 (2)	Cl1A—C11A—H11B	112.3
C9—N1—C5	118.7 (3)	H11A—C11A—H11B	109.9
C9—N1—Ru1	125.8 (2)	C11B^iii^—C11B—Cl1B	99 (9)
C5—N1—Ru1	115.4 (2)	C11B^iii^—C11B—H11C	111.9
O2—C1—O1	123.2 (3)	Cl1B—C11B—H11C	111.9
O2—C1—C2	118.0 (3)	C11B^iii^—C11B—H11D	111.9
O1—C1—C2	118.8 (3)	Cl1B—C11B—H11D	111.9
C1—C2—H2A	109.5	H11C—C11B—H11D	109.6
C1—C2—H2B	109.5		
			
O3—Ru1—O1—C1	90.1 (2)	Ru1^ii^—O2—C1—C2	177.8 (2)
O4^ii^—Ru1—O1—C1	−88.9 (2)	Ru1—O1—C1—O2	1.0 (4)
Ru1^ii^—Ru1—O1—C1	−0.2 (2)	Ru1—O1—C1—C2	−178.2 (2)
N1—Ru1—O1—C1	−177.8 (2)	Ru1^ii^—O4—C3—O3	−0.1 (4)
O1—Ru1—O3—C3	−91.2 (2)	Ru1^ii^—O4—C3—C4	179.3 (2)
O2^ii^—Ru1—O3—C3	87.8 (2)	Ru1—O3—C3—O4	1.0 (4)
Ru1^ii^—Ru1—O3—C3	−1.2 (2)	Ru1—O3—C3—C4	−178.5 (2)
N1—Ru1—O3—C3	173.6 (2)	C9—N1—C5—C6	1.9 (5)
O3—Ru1—N1—C9	41.4 (3)	Ru1—N1—C5—C6	−173.9 (3)
O1—Ru1—N1—C9	−48.5 (3)	N1—C5—C6—C7	0.2 (5)
O2^ii^—Ru1—N1—C9	131.3 (3)	N1—C5—C6—C10	178.1 (3)
O4^ii^—Ru1—N1—C9	−137.9 (3)	C5—C6—C7—C8	−2.0 (5)
O3—Ru1—N1—C5	−143.1 (2)	C10—C6—C7—C8	−179.8 (4)
O1—Ru1—N1—C5	127.0 (2)	C6—C7—C8—C9	1.6 (5)
O2^ii^—Ru1—N1—C5	−53.3 (2)	C5—N1—C9—C8	−2.3 (5)
O4^ii^—Ru1—N1—C5	37.6 (2)	Ru1—N1—C9—C8	173.0 (3)
Ru1^ii^—O2—C1—O1	−1.4 (4)	C7—C8—C9—N1	0.5 (6)

Symmetry codes: (i) −*x*, −*y*+1, −*z*; (ii) −*x*+1, −*y*+1, −*z*+1; (iii) −*x*, −*y*, −*z*+2.
